# A Memoir on a New Process for the Discovery of the Greater Part of Mineral Poisons, When Mingled with Coloured Liquids

**Published:** 1821-03

**Authors:** J. B. Orfila


### *A Memoir on a new Process for the Discovery of the greater Part of Mineral Poisons, when mingled with coloured Liquids.* By J. B. Orfila, M.D. &c.

I HAVE particularly insisted, in my work on Toxicology, on the difficulty often experienced in the discovery of poisonous substances of the mineral kind which have been mingled with coloured liquids, such as red wine, decoction of coffee, &c. Indeed, almost all the re-agents proper to render manifest a mineral poison dissolved in water, act in such a manner that we cannot recognize it when it is united with deep-coloured liquids. I may add, indeed, that, under certain circumstances, the precipitates produced by those tests are of such a colour, that, far from furnishing any sure indications respecting the nature of the poisonous substance, they lead us to believe that it does not exist in the deleterious mixture we examine. This assertion has been so well placed beyond doubt, by the numerous experiments detailed in my Treatise on Poisons, that it would be superfluous to adduce here any further examples for its support. Any one will be easily convinced, on reading what I have written on this subject: 1°, that a physician charged to make a report on a case of poisoning, would be blameable if he did not know the means for discovering poisonous substances mingled with coloured liquids; since, in the greater number of cases, those substances have been, either designedly or accidentally, mixed with the coloured drinks ordinarily used in domestic economy; 2°, that the processes hitherto known to determine beyond doubt the existence of mineral poisons in such mixtures, differ considerably for each of those poisons; 3°, that the chemical operations by which they are constituted, are almost always very complicated, and are often but very ill adapted for the fulfilment of the proposed object. Indeed, is it easy to demonstrate the presence of metallic arsenic, on calcining with potash and charcoal the dry product of the evaporation of a mixture of a grain of white oxide of arsenic and one or two pints of wine or decoction of coffee? Would similar measures furnish more readily the proof of the existence of a small quantity of corrosive sublimate, of verdigris, of muriate of tin, &c.? I believe not; and this object appears to me to be of so much importance, that I am induced to publish the results of a great number of experiments which I think calculated to simplify the solution of the problem in question. The following are the reasonings by which I was led to the discovery of the process which I am about to describe.

Mineral poisons mingled with deep-coloured liquids, act with the tests proper for their discovery in a different way from that in which they would act if they were dissolved in water: this circumstance depends entirely on the presence of the matter which colours the liquid mixed with the poison; on destroying, then, this colouring matter, the poison should act with the tests as if that matter were not present, provided that the agent employed to deprive the mixture of colour does not decompose the poisonous substance. Now, *chlorine dissolved in water* (oxymuriatic acid) possesses the power of depriving of their colour red wine, decoction of coffee and of tobacco, &c.* and it decomposes but a small number of the poisons of the mineral kind; and, consequently, might be advantageously employed. Experience has not failed to convince me that such was the case, and that the greater part of mineral poisons might be detected by those means.

*White oxide of arsenic.*—A mixture was made of a solution of white oxide of arsenic in water and red wine; a sufficient quantity of liquid and concentrated chlorine (strong oxymuriatic acid) was poured into it to cause it to assume a yellow colour; a reddish-yellow precipitate was formed, composed of chlorine and the glutinous matter contained in the wine: this precipitate was suffered to settle, and the liquor was filtered. A *white* precipitate was produced in the filtered liquor by lime-water, a *green* by the sulphate of ammoniated copper, and a *yellow* by hydro-sulphuric acid (sulphurated hydrogen gas dissolved in water): the presence of white oxide of arsenic was then demonstrated. It has sometimes happened that no precipitate has been obtained on mingling the tests just mentioned with the filtered liquor, because the quantity of oxide of arsenic contained in the mixture was too small, or because the chlorine, instead of being concentrated, was diffused in a large proportion of water. From these two circumstances it happened that the solution of oxide of arsenic was too weak to act with the tests in a sensible manner: in this case, it became

* In stating that chlorine deprives red wine, coffee, &c. of their colour, it is not meant that they render those liquids colourless, but only that it destroys their red or brown colour: indeed, the mixture obtained is of a yellow colour. This is of but little importance; for this shade of colour does not prevent the principal tests acting on mineral poisons as they would do if those poisons were merely dissolved in water.

necessary to evaporate the liquid, to concentrate it, and, when it has been reduced to a third of its original volume, it has constantly furnished the white, green, and yellow precipitates above mentioned. These considerations show that it is important that *concentrated and recently-prepared chlorine* should be employed in inquiries of this kind. Analogous experiments were made with white oxide of arsenic dissolved in water, and mixed with various quantities of decoction of coffee: the action of the chlorine and the tests was the same.

*Arsenic acid.*—When a mixture of red wine and arsenic acid is made, and it is discoloured by concentrated chlorine, a yellow precipitate is formed: if the liquor be filtered, it is found to redden the tincture of turnsole, and to give rise to a *white* precipitate with lime-water; a *light-blue* precipitate with the acetate of copper, especially if a few drops of chlorine are poured into the liquor; and a *rose-coloured* precipitate with the hydro-chlorate, but slightly acid, of cobalt: which prove that arsenic acid may be discovered by those tests as if it were merely dissolved in water. The same circumstances were observed when coffee was substituted for wine.

*Acid arseniate of potass.*—The mixture composed of this substance and red wine or decoction of coffee, may be readily discoloured by chlorine. The liquor when filtered, after the deposition of the precipitate, give rise to a *white* precipitate with lime-water, a *light-blue* with acetate of copper,* and a *rose-colour* with the hydro-chlorate, but slightly acid, of cobalt. If it should happen, by chance, that the last test does not produce any precipitate, the excess of its acid should be saturated with ammonia, (*liquor ammoniæ*, Pharm. Lond.) and the liquor will then soon be found to become opaque. It follows from those experiments, that chlorine will serve for the detection of the existence of arsenic acid and the soluble arseniates, when mingled with red wine, coffee, &c.

*Corrosive sublimate.*—A mixture of corrosive sublimate and wine was discoloured by *concentrated and recently prepared chlorine;* it was filtered after the precipitate of the chlorine and the vegeto-animal matter had settled; the filtered liquor

* Sometimes the precipitate, instead of being of a *light-blue* colour, is dark-blue, or a blue verging towards green: this happens when the quantity of chlorine employed for the discolouration of the wine or the coffee has not been sufficient. In this case, the colour of the precipitate may be changed to a light-blue by the addition of a few drops of chlorine. This circumstance has been observed in the greater part of the mixtures of poisons and coloured liquors which are considered in this memoir; so that I believe I may state, that in general it is sufficient to add a few drops of chlorine to the several precipitates obtained by means of the tests, to communicate to them the shade of colour which is proper to them, and that which they present when they result from the action of the test on the poison merely dissolved in water.

produced a *yellow* precipitate with potash, a *white* with ammonia, and a *black* with the hydro-sulphurets, as if the sublimate had been dissolved in water. A mixture was made of decoction of coffee and an aqueous solution of corrosive sublimate; the combination was treated with chlorine and the tests above mentioned, and precipitates of the same colour were obtained. In both cases, the liquor discoloured by chlorine has acted on a plate of copper deprived of its polished surface, as a solution of corrosive sublimate in water would do. It is, then, possible to discover the presence of this substance in wine and coffee, by the means which I have indicated. Nevertheless, I think it is preferable to have recourse to the following process: The mixture of sublimate and wine or coffee is put into a flask; two or three drachms of sulphuric ether are poured on it; the flask is to be corked, and gently shaken for ten or twelve minutes, so, however, that the ether may be several times in contact with every part of the liquor. The ether takes from the wine and the coffee the greater part of the sublimate, and the liquor separates into two *strata* when we cease to shake it: the upper stratum is formed by the ether holding in solution the corrosive sublimate. The whole is poured into a tunnel, the extremity of the pipe of which is closed by the fore-finger. After a few instants, when the separation of the liquor into the two strata above mentioned is seen to be effected in the body of the tunnel, the inferior coloured liquor is suffered to flow out, by partly removing the finger from the end of the pipe; when the lower stratum of the liquor is just flown out, the opening of the pipe of the tunnel is again closed by the finger, to prevent the escape of the etherial stratum, which is now to be poured into a cup or any other vessel presenting an extensive surface: the ether evaporates, and the sublimate is left in a solid state. This is then dissolved in a small quantity of distilled water, and a concentrated aqueous solution is thus obtained, in which the yellow, white, and black precipitates, which it furnishes with potash, ammonia, and the hydro-sulphurets, are easily recognized. The same process might be employed with success, where the sublimate has been dissolved in alcohol, or so large a quantity of water that it is in such a state impossible to detect its presence by the tests. Indeed, experience has proved to me that we may easily discover it when it exists in the proportion of only one grain to four thousand grains of distilled water. I think it useful to remark, that if the two liquors are shaken too violently, and for a very long time, and if the ether employed is not sufficient in quantity, the experiment will fail: indeed, the ether in this case would be entirely dissolved by the water or by the coloured liquor mixed with the poison, and we should not obtain the two strata of different specific gravity, on which the success of the whole operation rests.

It was known long since that corrosive sublimate was much more soluble in ether than in alcohol and in water. Mr. Chaussier announced, in 1811, from some experiments made by Mr. Henry, that ether took corrosive sublimate from water; but I do not know that any person has yet spoken of the experiments I have just related, relative to the use of ether for the separation of corrosive sublimate from coloured liquors which have not decomposed it.

*Acetate and sulphate of copper: artificial verdegris dissolved in water.*—Mixtures of red wine and various quantities of the salts just mentioned were made: chlorine dissolved in water and concentrated (strong oxymuriatic acid,) was added to the mixture, until it became of a yellow or greenish-yellow colour. A precipitate formed, composed of chlorine and vegeto-animal matter. The liquor was filtered: a *brown-marron* coloured precipitate was produced by the addition of prussiate of potash (hydrocyanate of potash and iron), a *green* by arsenite of potash, and a *black* by the hydro-sulphurets, or by the hydro-sulphuric acid. Now, these characters appertain to the salts of copper dissolved in water. Ammonia and potash acted on the mixture of wine and sulphate of copper discoloured by chlorine, as they do on the aqueous solution of sulphate of copper, though it was not thus, with a mixture of wine and acetate of copper, discoloured. This is of but very little importance, because the *brown-marron*, *green*, and *black* precipitates, obtained by the prussiate of potash, the arsenite of potash, and the hydro-sulphates, permit us to affirm that the solution contains a salt of copper. Sometimes the mixture of wine and cupreous salt, discoloured by chlorine, contains so small a quantity of the poison, that the tests do not render it manifest: the solution must here be concentrated by evaporation, as I stated when speaking of the oxide of arsenic. Coffee rapidly decomposes the salts of copper, and produces a precipitate so abundant, that it is impossible to suppose the possibility of such a mixture in the liquid state.

*Acid tartrate of potash and antimony,* (antimonium tartarizatum, *Ph. Lond.*)—Tartar emetic, being decomposed and precipitated by chlorine, should be ranged amongst the small number of poisons which do not remain in solution, when, after having been mingled with wine or coffee, they are treated with chlorine. The process under consideration is not, then, applicable to this particular case.

*Compositions of lead.*—If it be incontestible that the soluble salts of lead cannot be mingled with red wine or decoction of coffee, because they are decomposed by those liquids, it is equally true that, under certain circumstances, wine which has stood on litharge, or tobacco which has been packed in leaden boxes, combine with a proportion of lead sufficiently considerable to produce deleterious effects on our system: it is of importance, then, that the means for the detection of the saturnine preparation should be indicated.—*Red wine.* Acid wines which have been for some time in contact with litharge in small particles, may still preserve their red colour; they acquire an astringent and slightly sweet taste. When they are mixed with concentrated chlorine, they become discoloured; and, if they are filtered after the precipitate of chlorine and vegeto-animal matter which forms is suffered to settle, it is found that the liquor, when filtered, of a yellow colour, hardly furnishes, with the proper tests of lead, any sensible precipitate; the want of it depending on the small quantity of salt of lead contained in the wines. But, when the liquor is evaporated until it is reduced to one-third of its original volume, we *constantly* observe that the sulphate of potash gives rise to a *white* precipitate, the hydro-sulphates to a *black*, and chromate of potash a *bright-yellow;* from which it follows, that the process under consideration should be employed in preference to any other to demonstrate the presence of lead in wines imbued with litharge. The measure proposed by authors to accomplish this end, and which I had myself indicated, is certainly very conclusive, since it consists in separating the metallic lead by calcination, but it is a much longer and much more difficult process.—*Tobacco.* If tobacco has been packed in leaden boxes, and it contains oxide or subcarbonate of lead, it should be boiled for ten or twelve minutes with a mixture composed of equal parts of distilled water and distilled vinegar, to change the saturnine compound into a soluble acetate; it is to be filtered: the filtered liquor will be of so brown a colour, that it will not be possible to demonstrate the presence of the metal by the aid of the tests above mentioned; but this will be easily effected if the liquor is discoloured by means of chlorine, and treated as I have described when speaking of wines imbued with litharge.

*Acid nitrate of bismuth.*—The acid nitrate of bismuth may be united with such a quantity of wine, that the mixture may preserve the transparency and red colour of the wine: when a sufficient quantity of concentrated chlorine is poured into it, it becomes discoloured, and at the same time a precipitate of a yellowish-white colour is formed;* a few drops of hydro-chloric acid (muriatic acid) are sufficient to make the precipitate dis-

* Chlorine precipitates the acid nitrate of bismuth in a white powder.

appear, and then the liquor is of a yellow colour. Water and potash produce a *white* precipitate from it, though the precipitate formed by the alkali becomes yellow when it is dry; and the hydro-sulphates produce a *black* precipitate: the solution acts, then, with the tests as if the wine were not present. When mixed with coffee, the acid nitrate of bismuth is affected as it is when united with wine, when chlorine is added: however, the proportion of the coffee to the salt should not be very considerable, for there would then be formed a very abundant grumous precipitate.

*Sulphate of zinc.*—Red wine was mingled with a solution of sulphate of zinc in pure water; a quantity of concentrated solution of chlorine, sufficient to discolour it, was added: the precipitate of chlorine and vegeto-animal matter which then formed was permitted to settle, and the liquor was filtered: it was of a yellow colour, and furnished a *white* precipitate on the addition of potash, a *white* and *slightly-yellow* with the hydro-sulphurets. The tests, then, have acted on it as if the sulphate of zinc were dissolved only in water. A mixture was made of a decoction of coffee and a solution of pure sulphate of zinc: it was discoloured by means of chlorine; the liquor was filtered, and furnished a pale *yellowish-white* precipitate with the hydro-sulphurets, a *white* with the hydro-chlorate of barytes (muriate of barytes), and a *brown* with potash: but this last precipitate became *white* when a few drops of solution of chlorine (oxygenated muriatic acid) were poured on it.

*Hydro-chlorate of gold*, (muriate of gold.)—A mixture of red wine and hydro-chlorate of gold was discoloured by means of chlorine: the filtered liquor furnished a *deep-purple* precipitate with the proto-hydrochlorate of tin, a *deep-yellow* with ammonia, and a *blackish-brown* with the proto-sulphate of iron, (common sulphate of iron, or purified *green vitriol*.) The hydro-chlorate of gold, then, mingled with wine and discoloured by chlorine, acts with the tests as if it were simply dissolved in water. A mixture of coffee and the salt under consideration was discoloured by means of chlorine: the liquor, when filtered, furnished a *blackish-brown* precipitate with the proto-sulphate of iron, a *deep-purple* with the proto-hydrochlorate of tin, and a *deep-yellow* with ammonia: this last became of a *pale-yellow* when an excess of ammonia and a few drops of chlorine were added.

*Nitrate of silver.*—The nitrate of silver, being suddenly decomposed by chlorine, and transformed into an insoluble chloride of silver (oxy-muriate of silver), cannot be detected in the liquor when it has been mingled with wine or coffee and the mixture discoloured by means of chlorine.

*Deuto-hydrochlorate of tin.*—A mixture was made of this salt and red wine, and a quantity of chlorine added sufficient to discolour it: the precipitate formed was permitted to settle; the filtered liquor was yellow, and furnished a *white* precipitate with potash, and a *yellow* with the hydro-sulphurets, as if the deuto-hydrochlorate of tin had been simply dissolved in water. A mixture of this salt and coffee acted in the same way with chlorine and the tests.

*Proto-hydrochlorate of tin.*—When a mixture is made of proto-hydrochlorate of tin with wine, and concentrated solution of chlorine is added to discolour the liquor, it is found that this discolouration is very difficult to be effected: it does not take place until six times as much chlorine has been employed as is necessary for the destruction of the colour of wine mingled with the other mineral poisons; whence it appears that the proto-hydrochlorate of tin must be in but a small proportion to the liquor, and that the tests proper to detect it do not act on the mixture. But it is quite otherwise when the liquor is reduced to the fifteenth part of its volume by evaporation, and filtered: potash then gives rise to an abundant *white* precipitate, the hydro-sulphurets to a *yellow*, the infusion of cochineal to a *scarlet*, and the hydrochlorate of gold does not render it opaque or troubled. Now, these characters appertain to the proto-hydrochlorate of tin; whence we must conclude, that the chlorine has transformed the proto-hydrochlorate into a deuto-hydrochlorate, as if wine had not entered into the combination. This process, then, will enable the physician to affirm that the liquor contains a salt of tin (which is of the most importance), without permitting him to decide on the nature of the oxide which enters into the composition of this salt.

*Hydro-chlorate of barytes*, (muriate of barytes.)—When a concentrated solution of chlorine is poured into a mixture of red wine and hydrochlorate of barytes, or of this salt and decoction of coffee, the liquor is discoloured. If it is filtered after the precipitate of chlorine and the vegeto-animal matter has settled, we find that the filtered liquor, having also been heated so as to cause the excess of chlorine to be disengaged, furnishes, with nitrate of silver, a *white curdy* precipitate, composed of chloride of silver, insoluble in water and in nitric acid; and produces, with sulphate of soda, a *white* precipitate, composed of sulphate of barytes, insoluble in water and in nitric acid. The tests, then, act with this mixture as if the hydro-chlorate of barytes were alone present.

*Composition blue*, (a mixture of concentrated sulphuric acid and indigo.)—It is known that composition blue gives out sulphurous gas, recognizable by its odour, when it is boiled with metallic mercury; a proof that it contains sulphuric acid: but, if we wish to determine the presence of this acid, either by the tincture of turnsole or by the salts of barytes, we observe phenomena but little qualified to enlighten us in regard to the real nature of the liquor. It is not so when this is mixed with a concentrated solution of chlorine: discolouration of it instantly takes place, provided that a sufficient quantity of chlorine be employed, and the liquor resulting from that combination, of a yellow colour, gives a deep-red colour to the turnsole paper, and furnishes, with nitrate of barytes, a precipitate composed of sulphate of this oxide, which is of a white colour when it is collected into a heap, and which does not dissolve in either water or nitric acid. The tests act, then, as if indigo made no part of the mixture.

*Alum mingled with wine.*—It is well ascertained that wine-merchants sometimes add alum to red wine, to communicate to it a rough taste and a deeper colour: now, this mixture may produce in the system ill effects of a more or less serious kind. Several means have been proposed for the discovery of the fraud in question, but many of them might be shown to be ineffectual: none appears to me to be proper for the discovery of the presence of the alum but the following: The wine is to be discoloured by means of a concentrated solution of chlorine; the mixture is to be evaporated until it is reduced to nearly the fourth of its original volume; the liquor is to be filtered, and we then find it to possess the following properties when it contains alum:—1, it has a sweetish astringent taste; 2, it furnishes a *white* precipitate (sulphate of barytes) with nitrate of barytes, insoluble in water and in nitric acid; 3, caustic potash gives rise to a *yellowish-white* precipitate of alumine, soluble in an excess of potash; 4, the subcarbonate of soda produces a *yellowish-white* precipitate (subcarbonate of alumine), decomposable by fire into carbonic acid gas, and alumine easily recognizable by its characters.—(*Nouveau Journal de Médecine*, Juillet 1820.)

